# Recombinant protein expression in *Pichia pastoris *strains with an engineered methanol utilization pathway

**DOI:** 10.1186/1475-2859-11-22

**Published:** 2012-02-13

**Authors:** Florian W Krainer, Christian Dietzsch, Tanja Hajek, Christoph Herwig, Oliver Spadiut, Anton Glieder

**Affiliations:** 1 Graz University of Technology, Institute of Molecular Biotechnology, Graz, Austria; 2 Oliver Spadiut, Vienna University of Technology, Institute of Chemical Engineering, Research Area Biochemical Engineering, Gumpendorfer Strasse 1a, A-1060 Vienna, Austria; 3 Austrian Centre of Industrial Biotechnology (ACIB GmbH), Graz, Austria

**Keywords:** *Pichia pastoris*, methanol utilization pathway, Mut^+^, Mut^S^, recombinant protein expression, dihydroxyacetone synthase, formaldehyde dehydrogenase, transketolase, horseradish peroxidase, *Candida antarctica *lipase B

## Abstract

**Βackground:**

The methylotrophic yeast *Pichia pastoris *has become an important host organism for recombinant protein production and is able to use methanol as a sole carbon source. The methanol utilization pathway describes all the catalytic reactions, which happen during methanol metabolism. Despite the importance of certain key enzymes in this pathway, so far very little is known about possible effects of overexpressing either of these key enzymes on the overall energetic behavior, the productivity and the substrate uptake rate in *P. pastoris *strains.

**Results:**

A fast and easy-to-do approach based on batch cultivations with methanol pulses was used to characterize different *P. pastoris *strains. A strain with Mut^S ^phenotype was found to be superior over a strain with Mut^+ ^phenotype in both the volumetric productivity and the efficiency in expressing recombinant horseradish peroxidase C1A. Consequently, either of the enzymes dihydroxyacetone synthase, transketolase or formaldehyde dehydrogenase, which play key roles in the methanol utilization pathway, was co-overexpressed in Mut^S ^strains harboring either of the reporter enzymes horseradish peroxidase or *Candida antarctica *lipase B. Although the co-overexpression of these enzymes did not change the stoichiometric yields of the recombinant Mut^S ^strains, significant changes in the specific growth rate, the specific substrate uptake rate and the specific productivity were observed. Co-overexpression of dihydroxyacetone synthase yielded a 2- to 3-fold more efficient conversion of the substrate methanol into product, but also resulted in a reduced volumetric productivity. Co-overexpression of formaldehyde dehydrogenase resulted in a 2-fold more efficient conversion of the substrate into product and at least similar volumetric productivities compared to strains without an engineered methanol utilization pathway, and thus turned out to be a valuable strategy to improve recombinant protein production.

**Conclusions:**

Co-overexpressing enzymes of the methanol utilization pathway significantly affected the specific growth rate, the methanol uptake and the specific productivity of recombinant *P. pastoris *Mut^S ^strains. A recently developed methodology to determine strain specific parameters based on dynamic batch cultivations proved to be a valuable tool for fast strain characterization and thus early process development.

## Background

The methylotrophic yeast *Pichia pastoris *has become an important host organism for the high level production of recombinant proteins (e.g. [1-3]). It can be grown to high cell densities, is characterized by an efficient secretory system and many tools for molecular manipulation are available. Thus, *P. pastoris *has become an interesting and important alternative to bacterial expression systems such as *E. coli*, especially when it comes to the production of complex proteins which require typical eukaryotic post-translational modifications or contain disulfide bridges [4].

The ability of *P. pastoris *to utilize methanol as the sole carbon source is a crucial aspect of its metabolism. The enzyme alcohol oxidase (AOX, EC 1.1.3.13) catalyzes the first step in the recently described methanol utilization pathway (MUT pathway) [5]. The genome of *P. pastoris *contains two genes, *AOX*1 and *AOX*2, encoding two enzymes with AOX activity [2]. AOX1 can comprise up to 30% of the total soluble protein in extracts of *P. pastoris *grown solely on methanol [5-7], showing the outstanding strength of the AOX1 promoter (*p*AOX1). On the contrary, the second alcohol oxidase AOX2 is controlled by a much weaker promoter (*p*AOX2) and thus accounts for just 15% of the overall AOX activity in the cell [8]. The tight regulation of these promoters [9], the strong inducibility of *p*AOX1 and differently regulated *p*AOX1 promoter variants [10] as well as the alternative weaker AOX2-mediated methanol oxidation allow the design of different expression strains with specific properties. Three phenotypes of *P. pastoris *with regard to methanol utilization are currently available: I, Mut^+ ^(methanol utilization plus), where both *AOX *genes are intact and active; II, Mut^S ^(methanol utilization slow), where *AOX*1 is knocked out; and III, Mut^- ^(methanol utilization minus), which is unable to grow on methanol as the sole carbon source due to a knock-out of both *AOX *genes [2]. In Mut^+ ^and Mut^S ^strains the transcription of MUT pathway genes is repressed when grown in the presence of sufficiently high concentrations of glucose or glycerol. Employing either of the two natural AOX promoters, protein expression at high levels can be induced by methanol in media lacking such fermentable carbon sources [11]. De-repression at low concentrations of glucose or glycerol can be used for strong induction, if AOX1 promoter variants are used [10].

In general, Mut^+ ^strains are characterized by a higher growth rate than Mut^S ^strains and have also been reported to show higher productivities [12-15]. In addition, if antibiotics are used for selection of transformants, there is no direct need to employ Mut^S ^strains, but wildtype strains such as *P. pastoris *CBS7435 (which is identical to *P. pastoris *NRRL Y-11430 [16]) or *P. pastoris *X-33 can be used as hosts. This is why the majority of research so far has been performed with this phenotype. However, Mut^+ ^strains are very sensitive to transient high methanol concentrations, rendering the scale up of bioprocesses more difficult [15,17,18]. Methanol oxidation is also linked to hydrogen peroxide formation as a by-product which is known to cause cellular stress and to induce cell death [19]. The combustion of methanol (-727 kJ·C-mol^-1^) results in the production of heat, which might also constitute a problem in large scale processes. Another important issue is the high demand for oxygen in high cell density cultures of *P. pastoris *with Mut^+ ^phenotype [20,21]. By using *P. pastoris *strains with Mut^S ^phenotype these problems can be bypassed due to the lower methanol consumption rate. However, the Mut^S ^phenotype also leads to long induction times and reduced growth rates. Mixed feed strategies (e.g. glycerol and methanol) are commonly employed for the fermentation induction phase when using Mut^S ^strains. Hereby the cells are not dependent on the slow metabolization of methanol, which then primarily functions as an inducer, and can use the alternative C-source for growth [20,22]. Moreover, the strong production of AOX1 in Mut^+ ^strains during growth on methanol may compete with the production of recombinant proteins [15]. Other advantages and disadvantages of using the AOX1 promoter system in *P. pastoris *Mut^+ ^strains have been summarized by Macauley-Patrick et al. recently [7]. Interestingly, Mut^S ^strains have been reported to be advantageous over Mut^+ ^strains for the production of some recombinant proteins [23-25], which currently drives the ongoing discussion in the scientific community about the most favorable *P. pastoris *phenotype for recombinant protein production.

**Figure 1:**
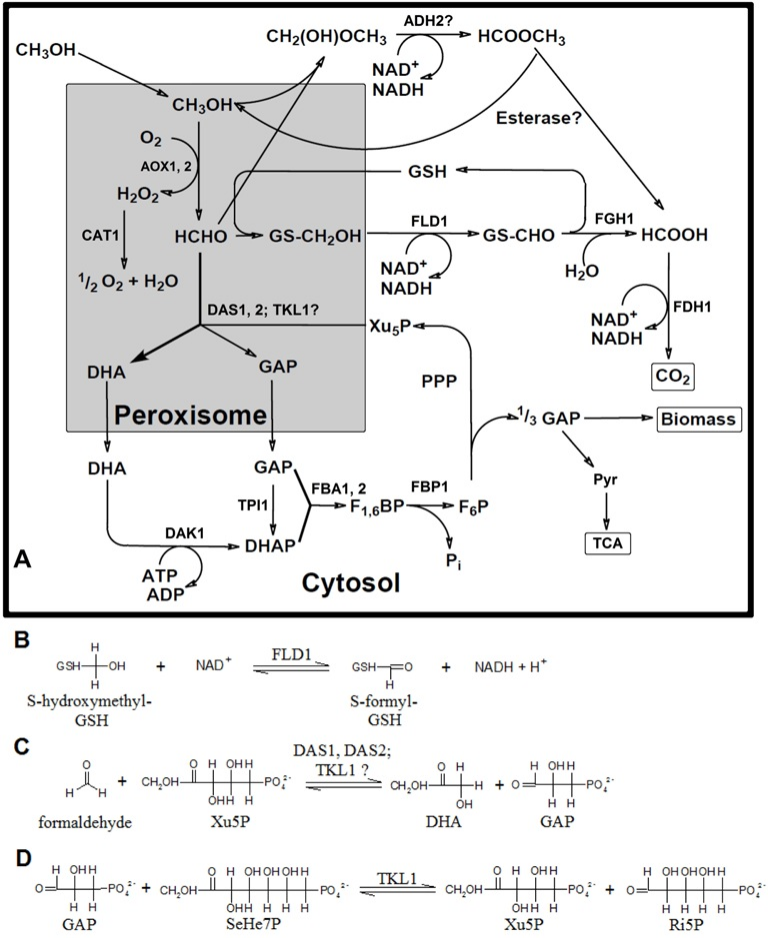
**Methanol utilization (MUT) pathway in *P. pastoris***. **A**, MUT pathway overview (adapted from [5] and [16]); **B**, catalytic reaction of DAS1, DAS2 and hypothetically TKL1; **C**, catalytic reaction of FLD1; **D**, catalytic reaction of TKL1 in the pentose phosphate pathway. ADH: methylformate synthase. AOX: alcohol oxidase. CAT: catalase. DAK: dihydroxyacetone kinase. DHA: dihydroxyacetone. DHAP: dihydroxyacetone phosphate. F_1,6_BP: fructose-1,6-bisphosphate. F_6_P: fructose-6-phosphate. FBA: fructose-1,6-bisphosphate aldolase. FBP: fructose-1,6-bisphosphatase. FLD: formaldehyde dehydrogenase. FDH: formate dehydrogenase. FGH: S-formylglutathione hydrolase. GAP: glyceraldehyde-3-phosphate. GSH: glutathione. Pyr: pyruvate. PPP: pentose phosphate pathway. Ri5P: ribose-5-phosphate. SeHe7P: sedoheptulose-7-phosphate. TCA: tricarboxylic acid cycle. Xu_5_P: xylulose-5-phosphate.

Regardless of which phenotype is used, AOX catalyzes the first reaction in the MUT pathway, oxidizing methanol to formaldehyde while reducing O_2 _to H_2_O_2 _(Figure 1A). Formaldehyde is then either oxidized to CO_2 _in the dissimilative branch of the MUT pathway giving 2 NADH molecules per molecule formaldehyde, or is condensed with xylulose-5-phosphate and subsequently converted to dihydroxyacetone and glyceraldehyde-3-phosphate in the assimilative branch of the MUT pathway (Figure 1A; see also [5]).

Knock-out studies of the genes encoding formaldehyde dehydrogenase (FLD, EC 1.2.1.1) and formate dehydrogenase (FDH; EC 1.2.1.2) revealed FLD, hereafter called FLD1, to be the key enzyme in the dissimilative branch of the MUT pathway [26,27]. FLD1 is encoded by the *FLD1 *gene and catalyzes the NAD^+^-dependent oxidation of S-hydroxymethylglutathione to S-formylglutathion (Figure 1B). Consequently, FLD1 has been studied in more detail in terms of being a promising enzyme for improved cofactor regeneration recently [28].

The key enzyme in the assimilative branch of the MUT pathway is dihydroxyacetone synthase (DAS; EC 2.2.1.3). In *P. pastoris*, two isoforms of DAS are encoded by the two genes *DAS1 *and *DAS2 *which are 91% identical [5,16]. Due to the high sequence identity, wrong sequences for these two genes were described before, which were artefacts from wrong assemblies during sequencing. Both DAS1 and DAS2 catalyze the conversion of xylulose-5-phosphate and formaldehyde to dihydroxyacetone and glyceraldehyde-3-phosphate (Figure 1C; [5,16]). Despite the importance of this enzyme, no analysis has been reported so far, describing whether the transcription of the two genes *DAS1 *and *DAS2 *is equally high induced upon methanol induction or if differences in transcription, such as for *AOX1 *and *AOX2*, occur.

Another important enzyme in the assimilative branch is the transketolase (TKL; EC 2.2.1.1) [16], hereafter called TKL1, which catalyzes the reaction between xylulose-5-phosphate and ribose-5-phosphate to form glyceraldehydes-3-phosphate and sedoheptulose-7-phosphate and vice versa (Figure 1D). In *Saccharomyces cerevisiae *transketolase activity has been shown to constitute the rate-limiting factor in the non-oxidative part of the pentose phosphate pathway [29], demonstrating the importance of this enzyme in yeast metabolism. In *P. pastoris *TKL1 has recently been assigned a hypothetical dihydroxyacetone synthase activity (Figure 1C; [16]). A highly conserved domain structure of the enzymes DAS1, DAS2 and TKL1 and the fact that a double knock-out strain of *DAS1 *and *DAS2 *is still able to grow on the substrate methanol (personal communication with Prof. Anton Glieder) actually underline this hypothesis (Additional file 1: Figure S1).

In this study, we determined and compared the specific substrate uptake rate (q_s_) and the specific productivity (q_p_) of a recombinant *P. pastoris *CBS7435 Mut^+ ^strain with a Mut^S ^strain, both overexpressing the reporter enzyme horseradish peroxidase (HRP), to determine the more efficient phenotype for recombinant protein expression. Based on our findings, we co-overexpressed either of the enzymes DAS1, TKL1 or FLD1, which all play key roles in the MUT pathway, in recombinant *P. pastoris *Mut^S ^strains harboring either of the reporter enzymes horseradish peroxidase (HRP) or *Candida antarctica *lipase B (CalB), to check for possible influences on stoichiometric yields, the growth rate, the methanol uptake and the production of the respective reporter enzyme. We used a novel and fast approach based on batch cultivations with repeated methanol pulses, which has proven to be a valuable alternative to the traditionally more often used strategies of either employing continuous cultures or repetitive fed-batch cultivations at different parameter sets [30,31], to determine these strain specific parameters.

## Results and discussion

### Mut^+ ^vs. Mut^S ^for recombinant protein production

To date, the majority of research has been performed with *P. pastoris *Mut^+ ^strains, since they have been reported to grow faster on methanol and thus to produce more recombinant protein (e.g. [14]). However, several other studies have shown Mut^S ^strains to be superior over Mut^+ ^strains in terms of recombinant protein production (e.g. [23]). To shed more light on this controversial topic, we designed *P. pastoris *strains overexpressing the reporter enzyme HRP in both phenotypes Mut^+ ^and Mut^S^, and compared these two strains in terms of specific substrate uptake rate, specific productivity and volumetric productivity. This strain characterization was performed using a recently reported strategy employing fast and easy-to-do batch cultivations with methanol pulses [30,31] and is shown for the Mut^S ^and for the Mut^+ ^phenotype in Additional file 2: Figure S2 and Additional file 3: Figure S3, respectively.

**Figure 2:**
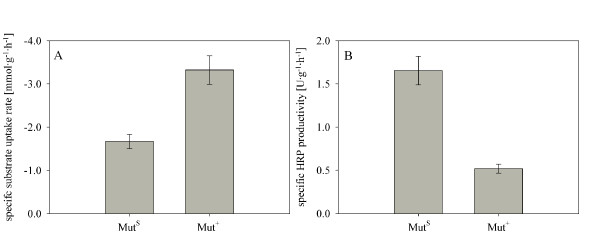
**Average values for specific rates obtained in pulse experiments with *P. pastoris *Mut^S ^and Mut^+ ^strains overexpressing HRP**. A, specific substrate uptake rate for methanol; B, specific HRP productivity.

The frequent determination of biomass, methanol and product concentration allowed specific rate calculations during methanol pulses. Average values for the specific rates were calculated out of several pulses to be able to compare the two strains on the basis of a reliable set of data. The specific growth rate of the Mut^+ ^strain was calculated to be approximately 1.5-fold higher than for the Mut^S ^strain (data not shown). As shown in Figure 2, the Mut^+ ^strain was also characterized by a nearly 2-fold higher specific uptake rate for methanol (q_s_), whereas the specific productivity (q_p_) was 3-fold lower compared to the Mut^S ^strain.

To combine the observed effect on q_s _and q_p _in just one parameter and thus put the productivity of the strains in direct relation to the consumed substrate, we introduced the efficiency factor η (Eq. 1), which is in fact the product yield respective to the substrate methanol.

(1) $$\eta =\left|\frac{q_{p}}{q_{s}}\right|{\;}_{\left[\text{U/mmol}\right]}$$

**Figure 3:**
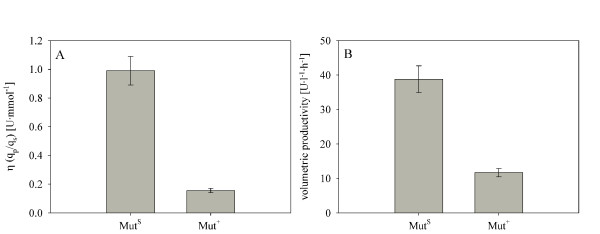
**Comparison of a *P. pastoris *Mut^S ^and Mut^+ ^strain overexpressing HRP**. A, efficiency factor η (relationship between q_p _and q_s_); B, volumetric productivity.

In Figure 3A, the benefit of using a Mut^S ^strain for the recombinant production of HRP is highlighted, as the substrate methanol was converted 7-fold more efficient into product compared to the Mut^+ ^strain.

Additionally, the volumetric productivity was calculated to compare the two Mut phenotypes in terms of product formation per volume and time. As shown in Figure 3B, the volumetric productivity of the Mut^S ^strain was more than 3-fold higher than for the Mut^+ ^strain, underlining the usefulness of this phenotype for the production of recombinant proteins. Interestingly, Morawski *et al*. reported up to 3-fold higher HRP activity levels using the *P. pastoris *wildtype strain X-33 with Mut^+ ^phenotype compared to the strain KM71 with Mut^S ^phenotype (*his4*, *aox1::ARG4*) [32]. An explanation for this contradictory result might be a difference in the gene copy number of HRP in the Mut^+ ^and Mut^S ^strains studied by Morawski *et al*. and/or differences in the operational conditions. Since the strains used in the present study both were found to have a single copy integration of the HRP encoding gene, and since the batch cultivations were performed in the same way in the same bioreactors, we can exclude such influences. Consequently, the *P. pastoris *Mut^S ^strain was chosen as the basis for subsequent overexpression studies.

### Transcription of the genes DAS1 and DAS2 upon methanol induction

In order to study a possible difference between *DAS1 *and *DAS2 *transcription upon methanol induction, as known for the *AOX1 *and *AOX2 *genes [33], we determined the respective transcript levels in a *P. pastoris *Mut^S ^strain and calculated the increase in transcription after 5 h upon induction. The transcription of *DAS1 *and *DAS2 *was found to be equally high induced upon methanol induction, as *DAS1 *transcription was 98.1-fold and *DAS2 *was 99.1-fold induced. Methanol induction of the *DAS1 *and *DAS2 *genes was also much stronger than for *AOX2 *(about 30-fold). Considering the high sequence similarity of *DAS1 *and *DAS2 *and the equivalent increase in transcript levels upon methanol induction, we focused on the co-overexpression of *DAS1 *to subsequently study the influence of emphasized dihydroxyacetone synthase activity on recombinant protein expression.

### Co-overexpression of the MUT pathway enzymes DAS1/FLD1/TKL1

**Figure 4:**
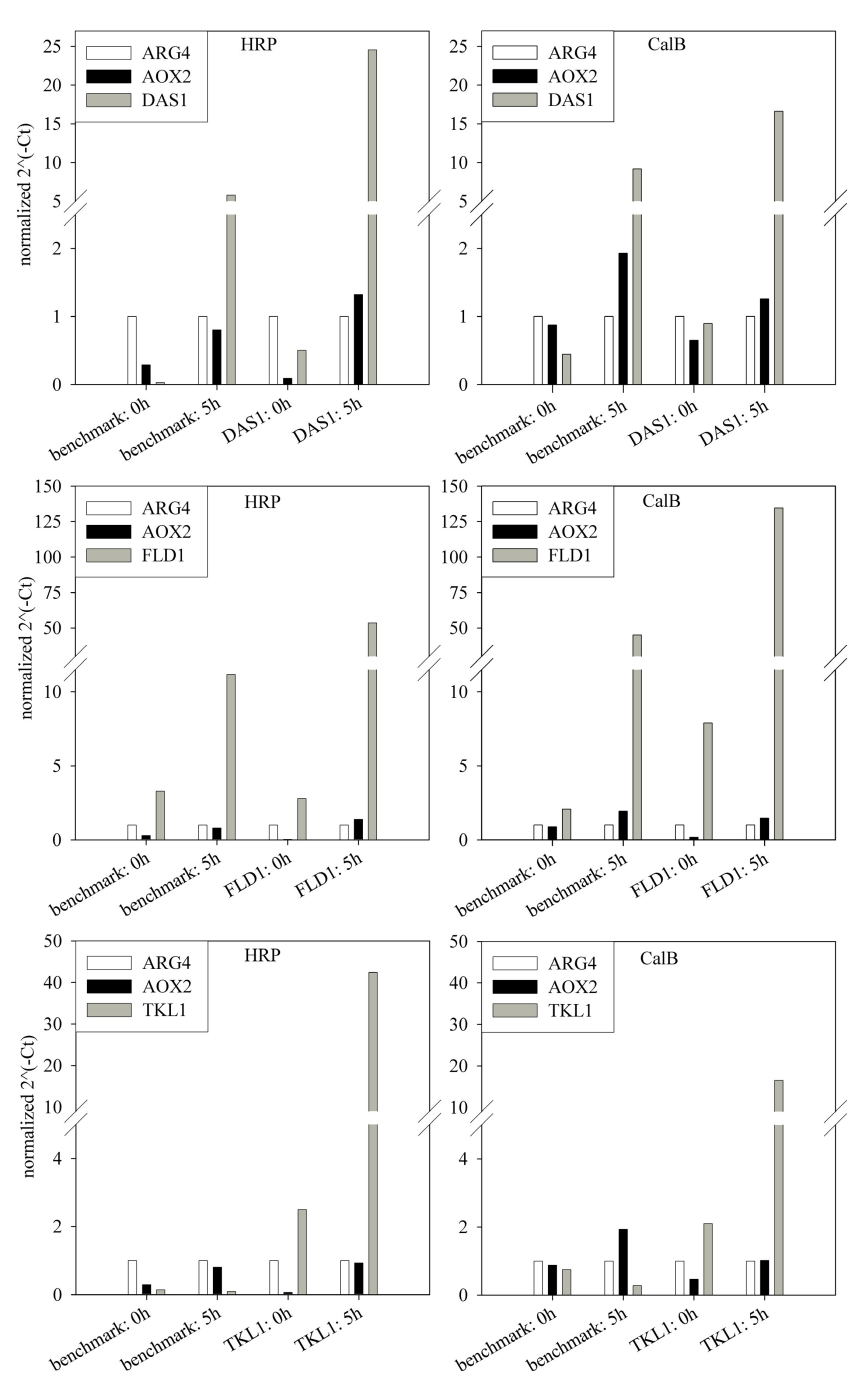
**Transcription analysis of the co-overexpressed MUT pathway genes *DAS1*/*FLD1*/*TKL1 *in HRP/CalB overexpressing strains**. The transcript levels were normalized to the corresponding transcript levels of *ARG4*. The increase in *AOX2 *transcript levels indicated successful induction with methanol. All co-overexpression strains showed elevated transcript levels of the respective target mRNAs compared to the benchmark strains.

We co-overexpressed three key enzymes of the MUT pathway, *i.e*. dihydroxyacetone synthase 1 (DAS1), formaldehyde dehydrogenase 1 (FLD1) and transketolase 1 (TKL1), to analyze possible effects on stoichiometric yields and on the strain specific parameters q_s _and q_p_. We used a Mut^S ^strain for this study, since it had been shown to be superior to the Mut^+ ^phenotype in terms of recombinant protein production (*vide supra*). To be able to draw more general valid conclusions from these experiments, we used two recombinant *P. pastoris *Mut^S ^strains overexpressing either the reporter enzyme HRP or CalB, hereafter called benchmark strains, as a platform for co-overexpression studies with the above mentioned MUT pathway enzymes. We characterized all strains in terms of gene copy numbers of the respective reporter enzyme to exclude variations in the measured enzyme activity levels due to copy number rearrangements, such as duplications or deletions. All the generated strains had a single copy integration of the gene encoding the respective reporter enzyme. Thus, all observed variations in enzymatic activity were considered to be due to the respective co-overexpressed MUT pathway enzyme. In order to verify the successful co-overexpression of the MUT pathway enzymes, the mRNA levels of *DAS1*, *FLD1 *and *TKL1 *were quantified relatively to the transcript levels of the constitutively transcribed *ARG4 *gene via qPCR analysis (Figure 4).

In all strains the transcript level of *AOX2 *increased upon methanol addition, indicating successful induction with methanol. We also observed elevated transcript levels of the respective mRNAs of *DAS1*, *FLD1 *and *TKL1 *upon methanol induction in all co-overexpression strains compared to the benchmark strains (Figure 4), proving the successful co-overexpression of the MUT pathway enzymes in these strains. However, transcript levels varied between HRP and CalB strains: the transcript level of *FLD1 *in the CalB FLD1 strain was 2.5-fold higher than in the HRP FLD1 strain, whereas *DAS1 *and *TKL1 *were 1.5-fold and 2.5-fold higher transcribed in HRP DAS1/TKL1 strains, respectively. This could be due to two reasons: despite the fact that the same amount of DNA was used for each transformation, different copy numbers of the respective genes might have integrated. Also, the genes might have integrated at different loci in the chromosome, which might influence the accessibility of the transcription machinery to the transformed gene and thus the extent of transcription. Regardless of these variations, all MUT pathway enzymes were successfully co-overexpressed, which is why observed effects on stoichiometric yields, the specific substrate uptake rate and the specific productivity of the recombinant *P. pastoris *Mut^S ^strains were ascribed to these enzymes.

### Characterization of P. Pastoris strains in the bioreactor

We characterized the *P. pastoris *Mut^S ^strains HRP FLD1/TKL1/DAS1 and CalB FLD1/TKL1/DAS1 using a fast and dynamic approach based on batch cultivations with repeated methanol pulses, as described in detail before [30,31].

#### Growth rate and stoichiometric yields of the different P. Pastoris strains

Due to the very low biomass growth rate of all *P. pastoris *strains in the pulsed batch experiments, the error of the dry cell weight duplicate measurements yielded standard deviations close to the determined biomass increase within one pulse (for an example see Additional files 2 and 3: Figures S2 and S3). Thus, the biomass growth rate had to be calculated using the carbon balance and the degree of reduction balance to get more accurate values. All the calculated h-values for a χ^2 ^test were lower than 3.84, implying that all determined values were within error margins. Furthermore, the respiratory quotients (RQ) of the strains were calculated using the online measured off-gas data and were compared with the theoretical values for RQ, which had been estimated based on the reconciled biomass yields. In general, calculated and theoretical RQ were very similar (data not shown), signifying that the reconciled biomass rates were accurate.

The specific growth rate was reduced 2- to 3-fold for strains which co-overexpressed either of the MUT pathway enzymes, compared to the benchmark strains. Apparently, the overexpression of yet another enzyme described an additional metabolic burden for the host cells and/or down-regulated the transcription of *AOX2*, as indicated by the transcription analysis (Figure 4), thus reducing the specific growth rate.

In analogy to the determination of the specific growth rates, we studied the influence of the co-overexpressed MUT pathway enzymes on the stoichiometric yields of the recombinant *P. pastoris *Mut^S ^strains. The yields of biomass on substrate (Y_X/methanol_), CO_2 _on substrate (Y_CO2/methanol_) and O_2 _on substrate (Y_O2/methanol_) were calculated in a way to close both the carbon balance and the degree of reduction balance to 100% [34]. No significant differences between the Mut^S ^strains could be observed as equal yields were detected for all the strains (*i.e*. Y_X/Methanol _= 0.42 C-mol·C-mol^-1^, Y_CO2/Methanol _= 0.57 C-mol·C-mol^-1 ^and Y_O2/Methanol _= 1.06 C-mol·C-mol^-1^). Apparently, the energetic behavior of the recombinant strains was not affected by co-overexpressing either of the three MUT pathway enzymes. This fact was further underlined by the analysis of the respiratory quotient (RQ), since no significant differences in RQ between the different strains were detected (Table 1).

**Table 1:** Summary Table.

reporter enzyme	MUT enzyme	RQ	**q**_**s**_ [mmol·g^-1^·h^-1^]	**q**_**p**_ [U·g^-1^·h^-1^]	η [U·mmol^-1^]	vol. productivity [U·l^-1^·h^-1^]
HRP	benchmark	0.54	- 1.67	1.66	0.99	38.79

	DAS1	0.56	- 0.51	1.56	3.06	28.35

	FLD1	0.57	- 1.22	2.01	1.65	49.11

	TKL1	0.57	- 0.88	1.96	2.22	39.87

CalB	benchmark	0.57	- 1.32	1.39	1.05	30.27

	DAS1	0.57	- 0.36	0.78	2.15	15.23

	FLD1	0.57	- 0.70	1.49	2.13	31.27

	TKL1	0.57	- 0.35	0.12	0.34	2.07

The respiratory quotient (RQ), the specific substrate uptake rate (q_s_), the specific productivity (q_p_), the efficiency factor (η) and the volumetric productivity of the benchmark strains (overexpressing either HRP or CalB) and the respective benchmark strains co-overexpressing either of the MUT pathway enzymes DAS1, FLD1 or TKL1 were determined

We believe that the fact that Mut^S ^strains metabolize methanol at a lower rate, and thus produce less of the intermediate formaldehyde, compared to Mut^+ ^strains, describes the metabolic bottleneck responsible for this outcome. The situation might be different for the Mut^+ ^phenotype, which has been found to be faster in methanol oxidation (*vide supra*). Thus, possible effects of overexpressing either of the MUT pathway enzymes, e.g. an increased formation of CO_2 _by overexpression of FLD1, have to be determined independently for Mut^+ ^strains.

#### Specific substrate uptake rate and specific productivity

The specific substrate uptake rate (q_s_) and the specific productivity (q_p_) of all the strains were calculated as average values from all the conducted pulses after data reconciliation and are summarized in Table 1.

Regarding q_s_, all strains co-overexpressing either of the MUT pathway enzymes consumed less methanol per biomass and time than the benchmark strains (Table 1). Co-overexpression of DAS1 decreased q_s _even up to 3-fold compared to the benchmark strains. Although the total numbers differ slightly the same trend for HRP strains and CalB strains can be seen (Table 1). The lowered q_s _might be explained by a general deceleration of the metabolism of the cells which have to deal with the overexpression of recombinant proteins (*i.e*. either of the reporter enzymes HRP or CalB and additionally either of the MUT pathway enzymes).

Interestingly, co-overexpression of FLD1 increased q_p _of both the HRP FLD1 strain and the CalB FLD1 strain compared to the benchmark strains, whereas co-overexpression of DAS1 decreased q_p _(Table 1).

Co-overexpression of TKL1 led to controversial results with regard to q_p_, as a slightly higher q_p _for HRP could be observed, whereas q_p _for CalB was even lower than for the benchmark strain. Since the strains were checked for the copy number of the integrated reporter enzyme and for the successful overexpression of the MUT pathway enzymes on transcript level (*vide supra*), and since the bioreactor cultivations of the TKL1 co-overexpressing strains were conducted repeatedly, giving the same results, we currently have no evident explanation for this outcome. We hypothesize that product specific effects might be the reason for the observed difference in q_p _for TKL1 co-overexpressing strains.

Due to the unaltered stoichiometric yields of the co-overexpression strains compared to the respective benchmark strains, we can currently not explain on which metabolic level the co-overexpressed MUT pathway enzymes affected the production of the recombinant reporter enzymes. However, this outcome underlines the importance and necessity of having a fast and easy-to-do methodology to characterize recombinant *P. pastoris *strains, as certain changes in the metabolism apparently might not have the same effect on different Mut^S ^strains harboring different target enzymes.

#### Efficiency factor η

Co-overexpression of DAS1 resulted in the most efficient conversion of the substrate methanol into product, regardless of which reporter enzyme was produced; for HRP 3-fold more product was obtained per substrate, and for CalB a more than 2-fold increase could be observed (Table 1). Also co-overexpression of FLD1 led to an approximately 2-fold increased efficiency for both reporter enzymes (Table 1). Interestingly, we found that the calculated efficiency factors correlated with the observed transcription levels of the co-overexpressed MUT pathway genes (Figure 4): *DAS1 *transcript levels were higher in the HRP strain than in the CalB strain, whereas *FLD1 *transcript levels were higher in the CalB strain than in the HRP strain. This trend can also be seen in the respective efficiency factors (Table 1). Thus, increasing the copy number of the respective MUT pathway gene in the genome of *P. pastoris *might intensify the beneficial effects on recombinant protein production and could be an interesting target for future studies.

For the recombinant strains co-overexpressing TKL1 different results for HRP strains and CalB strains were observed. While the co-overexpression of TKL1 resulted in a 2-fold more efficient conversion of methanol into the product HRP, a lower η than for the benchmark strain was detected for the production of CalB due to the very low q_p _(Table 1).

Summarizing, all strains which co-overexpressed enzymes of the MUT pathway, excluding the CalB TKL1 strain, showed a more efficient conversion of the substrate methanol into the respective product. In particular, an expression platform, where the MUT pathway enzyme DAS1 is overexpressed, could be interesting for industrial large-scale protein production processes due to significantly lower substrate consumption and consequently a reduced risk management compared to strains without an engineered MUT pathway.

#### Volumetric productivity

Besides the importance of reducing the required amount of methanol and the associated reduction in cost and risk management, the volumetric productivity, *i.e*. the amount of product per volume and time, is an important factor in industrial production processes. The determined average volumetric productivities of the different recombinant *P. pastoris *strains are summarized in Table 1.

In terms of volumetric productivity only FLD1 co-overexpressing strains showed slightly higher or similar volumetric productivities compared to the benchmark strains, whereas the DAS1/TKL1 co-overexpressing strains produced less product per volume and time. The beneficial effect of co-overexpressing the MUT pathway enzyme DAS1, which resulted in a much better conversion of substrate to product (Table 1), obviously came at cost of process time.

Taking into account all the different strain specific parameters, an expression platform where the MUT pathway enzyme FLD1 is overexpressed turned out to be a very interesting tool for the industrial production of proteins, since not only methanol can be converted into product 2-fold more efficiently, but also the volumetric productivity is at least equally high compared to benchmark strains.

In summary, several aspects have to be considered when designing and choosing a production strain, *i.e*. if the main goal is a significant reduction in cost and risk management due to a more efficient conversion of substrate into product or a fast production process due to a high volumetric productivity. With the methodology described here, these strain characteristics can be determined in a fast and easy way, which significantly speeds up bioprocess development.

## Conclusions

In this study a fast and easy-to-do method based on batch cultivations with methanol pulses was used to characterize different recombinant *P. pastoris *strains. The results can be summarized as follows:

• a direct comparison of a Mut^S ^and a Mut^+ ^strain, both overexpressing the recombinant enzyme HRP, revealed the Mut^S ^strain to be 7-fold more efficient in the conversion of substrate into product and to have a 3-fold higher volumetric productivity.

• the transcription of the genes *DAS1 *and *DAS2*, which encode the key enzymes of the assimilative branch of the MUT pathway, was equally high induced upon MeOH addition.

• co-overexpression of either of the MUT pathway enzymes DAS1, TKL1 or FLD1 in *P. pastoris *Mut^S ^strains overexpressing either HRP or CalB did not cause any changes in the energetic behavior of the strains, as calculated yields and observed respiratory quotients were equal, but significantly reduced the growth rate.

• co-overexpressing MUT pathway enzymes affected the specific substrate uptake rate as well as the specific productivity of recombinant Mut^S ^strains. The co-overexpression of DAS1 resulted in a 2- to 3-fold more efficient conversion of methanol into product, but came at cost of process time.

• co-overexpression of FLD1 resulted in an approximately 2-fold increased efficiency in the conversion of methanol into product and showed at least equally high volumetric productivities compared to benchmark strains, making this expression platform highly interesting for industrial production processes.

• since product specific effects might influence certain strain specific parameters, as shown for TKL1 overexpressing strains in this study, the necessity of having a fast methodology to characterize recombinant strains before going into industrial production processes was highlighted. The methodology described here provides such a tool and has great potential for the use in early process development in an industrial environment.

### Outlook

The present study shows the effect of co-overexpressing MUT pathway enzymes on strain specific parameters of different recombinant *P. pastoris *Mut^S ^strains and revealed interesting targets for strain engineering. However, this study was performed using only a Mut^S ^strain as expression platform since, it showed significantly better properties compared to the Mut^+ ^strain (without co-overexpression of MUT pathway genes). Thus, effects on some strain specific parameters might be more pronounced in Mut^+ ^strains and have to be evaluated independently. Moreover, the molecular mechanisms that cause the beneficial influence of the studied MUT pathway enzymes on the efficiency in recombinant protein production remain to be elucidated in more detail. Increasing the copy number of the respective MUT pathway genes in the chromosome might further intensify their effects and should be considered when designing a specific *P. pastoris *expression host in future studies. We further recommend to analyze interesting co-overexpressing strains in fed-batch cultivations, which can be easily set up with the parameters extracted out of the described batch method with methanol pulses (see also [30,31]), to test their long term stability in production processes.

## Material and methods

### Chemicals

Enzymes were purchased from Fermentas GmbH, Germany. 2,2'-azino-bis(3-ethylbenzthiazoline-6-sulphonic acid) diammonium salt (ABTS) was obtained from Sigma-Aldrich Handels GmbH, Austria. Difco™ yeast nitrogen base w/o amino acids (YNB), Bacto™ tryptone and Bacto™ yeast extract were obtained from Becton Dickinson and Company, Austria. Zeocin™ was obtained from InvivoGen-Eubio, Austria. D-Biotin and p-nitrophenyl butyrate (p-NPB) were obtained from Fluka Chemia AG, Switzerland. All other chemicals were purchased from Carl Roth GmbH & Co. KG, Germany.

### Strains and vectors

The strain *P. pastoris *Mut^S ^(Δ*aox*1::*FRT*) was engineered by Näätsaari *et al*. (manuscript in preparation) at Graz University of Technology, Institute of Molecular Biotechnology, based on the *P. pastoris *wildtype strain CBS7435. These two strains were used as starting strains for the corresponding overexpression studies. The shuttle vectors pPpT4_S and pPpKan_S, derivatives of pPpT2 [35] with two point mutations in the EM72 promoter and a *Smi*I restriction site instead of *Bgl*II, were used for cloning. pPpKan_S contains a kanamycin/geneticin resistance gene instead of a zeocin resistance gene [36,37]. pPpT4_S was used for harboring either the HRP isoenzyme C1A or CalB, which both were codon-optimized for high-level expression in *P. pastoris*. The codon table described in [35] was applied for codon optimization. Overexpression of *DAS*1, *FLD*1, *TKL*1 and the genes encoding HRP and CalB was under the control of *p*AOX1. Secretion of HRP and CalB to the cultivation supernatant was facilitated by a N-terminally fused prepro-signal sequence of the *S. cerevisiae *alpha-factor. pPpKan_S was used as shuttle vector harboring *DAS1*, *FLD1 *or *TKL1*, which were amplified from chromosomal DNA of *P. pastoris *CBS7435 using the cloning primers depicted in Table 2A with Phusion™ high fidelity DNA-polymerase (Finnzymes Oy, Finland) and GC buffer according to the manufacturer's protocol.

**Table 2:** Oligonucleotide-primers used for A, amplification of *DAS1*/*FLD1*/*TKL1 *from chromosomal DNA; B, evaluation of *DAS1*/*DAS2 *transcription induction; C, the analysis of *DAS1*/*FLD1*/*TKL1 *overexpression; D, copy number determination of the reporter genes (via verification of the Zeo^R ^copy number)

*A*.	*target*	*orientation*	*sequence 5'-3'*
	DAS1	forward	AAAAGGCGCGCCGAAACGATGGCTAGAATTCCCAAAGCAG

		reverse	TTTGCGGCCGCTTACAACTTGTCATGCTTTGGTTTTC

	FLD1	forward	AACACTAGTATGTCTACCGAAGGTCAA

		reverse	AACGCGGCCGCTTAGTGCATAGTAATCAC

	TKL1	forward	AGAGAATTCGAAACGATGTCTGATCTCTTAGCTATCAACAC

		reverse	AGAGCGGCCGCCTACGCATGAACAGACTCAAAAG

***B*.**	***target***	***orientation***	***sequence 5'-3'***

	ARG4	forward	TGCTGGCTACAGATCTTGCCGACT

		reverse	CTCGGCTTGTCTGACACATTCACCAG

	AOX2	forward	ATACTCATCCGAGGCCAGAGCTTACG

		reverse	ACCGTGAGCAAGACCAGCAGTCAA

	DAS1	forward	CTGAGAAACCAGCTAAAGGTGACGAGT

		reverse	TCTTGTCCCTCACGAGGGTACTCT

	DAS2	forward	CTGAAAAACCAGCCGAGGGTGATC

		reverse	TTCCTCACCTTCTTGAGGATAGTTCTTAACG

***C*.**	***target***	***orientation***	***sequence 5'-3'***

	ARG4	forward	TGCTGGCTACAGATCTTGCCGACT

		reverse	CTCGGCTTGTCTGACACATTCACCAG

	AOX2	forward	ATACTCATCCGAGGCCAGAGCTTAC

		reverse	ACCGTGAGCAAGACCAGCAGTCAA

	DAS1	forward	CTGAGAAACCAGCTAAAGGTGACGAGT

		reverse	TCTTGTCCCTCACGAGGGTACTCT

	FLD1	forward	TGGATTATCTGTCATCCAAGGTGCAGTTTC

		reverse	GTCCGCCCATGCCTTCTTTGAATC

	TKL1	forward	GTCGCTACACATGACTCGATTGGTC

		reverse	CATGAGGTTTGGAAGAGCTCTCAAGTG

***D*.**	***target***	***orientation***	***sequence 5'-3'***

	Zeo^R^	forward	GACTCGGTTTCTCCCGTGACT

		reverse	CTGCGGAGATGAACAGGGTAA

	ARG4	forward	TCCTCCGGTGGCAGTTCTT

		reverse	TCCATTGACTCCCGTTTTGAG

### Strain construction and screening procedure

Transformation of approximately 2 μg *Smi*I-linearized pPpT4_S or pPpKan_S plasmid DNA harboring the respective gene of interest into *P. pastoris *was done as described by Lin-Cereghino *et al*. [38]. Selection of successfully transformed clones was performed on YPD agar plates containing 25 mg·l^-1 ^zeocin (for HRP/CalB strains) or 25 mg·l^-1 ^zeocin plus 300 mg·l^-1 ^geneticin (for HRP/CalB DAS1/FLD1/TKL1 strains). Screening of randomly chosen transformants and rescreening of promising clones (in quadruplicates) were done as micro-scale cultivations in 96-deep well plates similar to the protocol described by Weis *et al*. [39]: cells were cultivated in 250 μl BMD1% (1% α-D(+)-glucose monohydrate, 6.0 g·l^-1 ^K_2_HPO_4_, 23.6 g·l^-1 ^KH_2_PO_4_, 13.4 g·l^-1 ^YNB, 0.4 mg/l D-biotin) for approximately 60 h, followed by addition of 250 μl BMM2 (1% methanol, 6.0 g·l^-1 ^K_2_HPO_4_, 23.6 g·l^-1 ^KH_2_PO_4_, 13.4 g·l^-1 ^YNB, 0.4 mg·l^-1 ^D-biotin) and 50 μl BMM10 (5% methanol, 6.0 g·l^-1 ^K_2_HPO_4_, 23.6 g·l^-1 ^KH_2_PO_4_, 13.4 g·l^-1 ^YNB, 0.4 mg·l^-1 ^D-biotin) per well 12 h, 24 h and 36 h after the first addition of BMM2.

HRP activity was verified by using an ABTS assay: after centrifugation of the induced transformants (3000 g, 10 min), 15 μl of the cultivation supernatant were mixed with 140 μl assay solution (0.5 mM ABTS in 50 mM NaOAc, pH 4.5, 2.9 mM H_2_O_2_) in a 96-well PS-microplate [32]. The increase in absorbance at 405 nm was followed in a Spectramax Plus 384 platereader (Molecular Devices, Germany) at room temperature for 5 min. CalB activity was evaluated by an esterase activity assay similar to the assay described by Zhang et al. [40]: 20 μl cultivation supernatant were mixed with 180 μl fresh assay solution (4 mM p-NPB in 300 mM Tris-HCl, pH 7.4, 1% dimethyl sulfoxid). The increase in absorbance at 405 nm was followed in a Spectramax Plus 384 platereader at room temperature for 5 min.

Evaluation of the transcription of *DAS1 *and *DAS2 *upon methanol induction was performed by quantitative real-time PCR (qPCR). For this purpose, RNA was isolated from the corresponding strains before and 5 h after methanol induction using the ZR Fungal/Bacterial RNA MicroPrep™ kit from Zymo Research Corporation (Irvine, CA, USA). In-column DNase digestion was performed according to the manufacturer's recommendations using RNase-free DNase I from Zymo Research. RNA was eluted in 20 μl DNase/RNase-free water (plus 1 μl RNaseOUT™ Recombinant Ribonuclease Inhibitor from Invitrogen Corporation, CA, USA) and used directly for qPCR using the SuperScript^® ^III Platinum^® ^SYBR^® ^Green One-Step qRT-PCR Kit (Invitrogen). The qPCR was performed in the ABI PRISM 7500 Real Time PCR System (Applied Biosystems, CA, USA) according to the manufacturer's recommendations employing the primers listed in Table 2B. For visualization of the magnitude of transcription induction, the determined cycle threshold (Ct) signals were transformed to 2^-Ct ^and normalized to the corresponding *ARG4 *signals (which is supposed to be constitutively transcribed). The extent of induction of a target transcript was calculated as the ratio of the normalized 2^-Ct ^signal of the target transcript at 5 h over the normalized signal at 0 h (*i.e*. before induction). The time point 0 h represented the de-repression state of the *P. pastoris *culture, since the C-source glucose was depleted before the addition of methanol. Analogically, the overexpression of genes encoding MUT pathway enzymes was verified via qPCR with the primers listed in Table 2C. The transcript levels were shown as 2^-Ct ^signals, normalized to the corresponding *ARG4 *signals.

To determine the copy number of the transformed genes, a qPCR based method was used as described by Abad *et al*. [41] using the Power SYBR^® ^Green Master Mix (Applied Biosystems) with the ABI PRISM 7500 Real Time PCR System. Hereto, genomic DNA was isolated from *P. pastoris *according to the protocol by Hoffman *et al*. [42]. The primers (Table 2D) were used at concentrations of 200 nM per primer with 0.66 ng of genomic DNA as template. The copy number of the genes encoding the target enzymes was determined indirectly via verification of the copy number of the Zeocin™ resistance-mediating gene Zeo^R^. Conditions were 10 min at 95 C, 40 cycles of 15 s at 95 C and 60 s at 60 C followed by a final dissociation step.

### Strain characterization in bioreactors

#### Culture media

Precultures were done in yeast nitrogen base medium (YNBM; 0.1 M potassium phosphate buffer pH 6.0, 3.4 g·l^-1 ^YNB w/o amino acids and ammonia sulfate, 10 g·l^-1 ^(NH_4_)_2_SO_4_, 400 mg·l^-1 ^biotin, 20 g·l^-1 ^glucose). Batch cultivations were performed in basal salt medium (26.7 ml·l^-1 ^85% phosphoric acid, 1.17 g·l^-1 ^CaSO_4_·2H_2_O, 18.2 g·l^-1 ^K_2_SO_4_, 14.9 g·l^-1 ^MgSO_4_·7H_2_O, 4.13 g·l^-1 ^KOH, 44 g·l^-1 ^C_6_H_12_O_6_·H_2_O, 0.2 ml·l^-1 ^Antifoam Struktol J650, 4.35 ml·l^-1 ^PTM1, NH_4_OH as N-source). Trace element solution (PTM1) was made of 6.0 g·l^-1 ^CuSO_4_·5H_2_O, 0.08 g·l^-1 ^NaI, 3.0 g·l^-1 ^MnSO_4_·H_2_O, 0.2 g·l^-1 ^Na_2_MoO_4_·2H_2_O, 0.02 g·l^-1 ^H_3_BO_3_, 0.5 g·l^-1 ^CoCl_2_, 20.0 g·l^-1 ^ZnCl_2_, 65.0 g·l^-1 ^FeSO_4_·7H_2_O, 0.2 g·l^-1 ^biotin, 5 ml·l^-1 ^H_2_SO_4_. Induction was carried out in presence of 1 mM δ-aminolevulinic acid for HRP overexpressing strains. The concentration of the base NH_4_OH was determined by titration with 0.25 M potassium hydrogen phthalate.

#### Experimental procedure

##### Preculture

Frozen stocks (-80 C) were pre-cultivated in 100 ml YNBM in 1000 ml shake flasks at 28 C and 230 rpm for max. 24 h. The grown preculture was transferred aseptically to the respective culture vessel. The inoculation volume was approximately 10% of the final starting volume.

##### Batch cultivation

Batch cultivations were carried out in either a 3 l or a 5 l working volume glass bioreactor (Infors, Switzerland). Basal salt medium was sterilized in the bioreactor and pH was adjusted to pH 5.0 by using concentrated NH4OH solution after autoclaving. Sterile filtered trace elements were transferred to the reactor aseptically. Dissolved oxygen (dO2) was measured with a sterilizable dO_2 _electrode (Visiferm™, Hamilton, Switzerland). The pH was measured with a sterilizable electrode (Easyferm™, Hamilton, Switzerland) and maintained constant with a PID controller using NH_4_OH solution (1 to 3 M). Base consumption was determined gravimetrically. Cultivation temperature was set to 28 C and agitation was fixed to 1200 rpm. The culture was aerated with 1.0 vvm dried air and off-gas of the culture was measured by using an infrared cell for CO_2 _and a paramagnetic cell for O_2 _concentration (Servomax, Switzerland). Temperature, pH, dO_2_, agitation as well as CO_2 _and O_2 _in the off-gas were measured online and logged in a process information management system (PIMS; Lucullus, Biospectra, Switzerland).

After the complete consumption of the substrate glucose, indicated by an increase of dO_2 _and a drop in off-gas activity, the first methanol pulse of a final concentration of 0.5% (v/v) was conducted with methanol (supplemented with 12 ml·l^-1 ^PTM1). Following pulses were performed with 1% methanol (v/v). All pulses were conducted directly after exhaustion of the substrate without lack phases. For each pulse, at least two samples were taken to determine the concentrations of substrate and product, as well as dry cell weight to calculate specific rates and yields.

#### Analysis of growth- and expression-parameters

Dry cell weight was determined by centrifugation of 5 ml culture broth (5000 rpm, 4 C, 10 min) in a laboratory centrifuge (Sigma 4 K15, rotor 11156), washing the pellet with 5 ml deionized water and subsequent drying at 105 C to a constant weight in an oven.

The enzymatic activity of HRP was measured using an ABTS assay in a CuBiAn XC enzymatic robot (Innovatis, Germany). Ten μl of sample were mixed with 140 μl 1 mM ABTS solution (50 mM KH_2_PO_4_, pH 6.5). The reaction mixture was incubated at 37 C for 5 min before the reaction was started by the addition of 20 μl 0.078% H_2_O_2 _(v/v). Changes in absorbance at 415 nm were measured for 80 s and rates were calculated. The standard curve was prepared using a commercially available HRP preparation (Type VI-A, Sigma-Aldrich, USA) in the range from 0.02 to 2.0 U·ml^-1^.

The enzymatic activity of CalB was measured by determining the esterase activity of CalB. A stock solution was generated by mixing 42 μl p-NPB with 458 μl DMSO and stored at -20 C. The assay reagent was prepared freshly by addition of 100 μl stock solution to 10 ml TrisHCl (300 mM, pH 7.5). Color development at 405 nm was followed in a UV-1601 spectrophotometer (Shimadzu GmbH; Austria) at 30 C for 5 min. One Unit of CalB was defined as the formation of 1 μmol p-nitrophenol·min^-1^.

#### Substrate concentrations

Concentrations of methanol were determined in cell free samples by HPLC (Agilent Technologies, USA) equipped with an ion-exclusion column (Supelcogel C-610H Sigma-Aldrich, USA) and a refractive index detector (Agilent Technologies, USA). The mobile phase was 0.1% H_3_PO_4 _with a constant flow rate of 0.5 ml·min^-1 ^and the system was run isocratically. Calibration was done by measuring standard points in the range from 0.1 to 10 g·l^-1 ^methanol.

#### Data analysis

Measurements of biomass concentration, product concentration and substrate concentration were executed in duplicates. Along the observed standard deviation for the single measurements, the error was propagated to the specific rates q_s _and q_p _as well as to the yield coefficients. The error for the volumetric rate of product formation was set to 5%.

#### Data reconciliation

The Mut^S ^phenotype caused a slow biomass growth during conducted methanol pulses. Thus, the determined increase of biomass during one pulse was close to the standard deviation observed for one duplicate measurement and could therefore not be regarded as accurate. Hence, the biomass rate was calculated using the MATLAB software (MathWorks, USA), using the carbon balance and the degree of reduction balance, made of the determined volumetric rates for the substrate uptake, the oxygen consumption and the carbon dioxide evolution. As the system of equations consisted of two elemental balances and only one rate to be calculated, the degree of freedom was 1. Consequently, the statistical test value h had to be lower than 3.84 not to reject the null hypothesis, that there were no errors in the measurements at a 95% confidence level for each data point. Including error margins of 5% for off-gas measurements and substrate determinations, a χ^2 ^(chi-square distribution) test was conducted for each data point and gave the corresponding h-values. This method was described in detail elsewhere [34].

## Abbreviations

ADH: methylformate synthase; AOX: alcohol oxidase; CalB: *Candida antarctica *lipase B; CAT: catalase; CER: carbon dioxide emission rate; C-mol: molarity of component based on one carbon atom; DAK: dihydroxyacetone kinase; DAS1: dehydroxyacetone synthase 1; DHA: dihydroxyacetone; DHAP: dihydroxyacetone phosphate; F_1,6_BP: fructose-1,6-bisphosphate; F_6_P: fructose-6-phosphate; FBA: fructose-1,6-bisphosphate aldolase; FBP: fructose-1,6-bisphosphatase; FDH: formate dehydrogenase; FGH: S-formylglutathione hydrolase; FLD1: formaldehyde dehydrogenase; GAP: glyceraldehyde 3-phosphate; GSH: glutathione; HRP: horseradish peroxidase; MUT: methonal utilization pathway; Mut^+^: methanol utilization positive (*AOX*1 intact); Mut^-^: methanol utilization minus (knock-out of *AOX1 *and *AOX2*); Mut^S^: methanol utilization slow (knock-out of *AOX*1); OUR: oxygen uptake rate; PPP: pentose phosphate pathway; Pyr: pyruvate; q_p_: specific productivity [U·g^-1^·h^-1^]; q_s_: specific substrate uptake rate [mmol·g^-1^·h^-1^]; Ri5P: ribose-5-phosphate; SeHe7P: sedoheptulose-7-phosphate; TCA: tricarboxylic acid cycle; TKL1: transketolase; Xu_5_P; xylulose-5-phosphate; η: efficiency factor.

## Competing interests

The authors declare that they have no competing interests.

## Authors' contributions

FK, CD, TH and OS designed and performed the experiments, analyzed and interpreted the data. FK, CD and OS wrote the manuscript. AG, CH and OS conceived the study and supervised the research. All authors read and approved the final manuscript.

## Supplementary Material

Additional file 1 **Figure S1**. Domain structure of DAS1, DAS2 and TKL1 (conserved domain prediction via CD-search tool [43-45]). All three enzymes belong to I, a thiamine pyrophosphate (TPP)-enzyme superfamily [NCBI CDD:cl01629]; II, a superfamily of TPP-depending enzymes containing a pyrimidine binding domain [NCBI CDD:cl11410]; and III, a superfamily of enzymes with a transketolase C-terminal domain [NCBI CDD:cl08363]. Amino acid sequences of *P. pastoris *strain CBS7435 from [16]. Click here for file

Additional file 2 **Figure S2**. Experimental strategy for the fast determination of strain specific parameters of the *P. pastoris *Mut^S ^HRP strain using a batch experiment with methanol pulses of 0.5% and 1% (v/v). A, (continuous line), oxygen uptake rate OUR; (diamond) biomass dry cell weight concentration; B, (continuous line), carbon dioxide emission rate CER; (circle), calculated specific substrate uptake rate q_s_; (triangle up), calculated specific HRP productivity q_p._ Click here for file

Additional file 3 **Figure S3**. Experimental strategy for the fast determination of strain specific parameters of the *P. pastoris *Mut^+ ^HRP strain using a batch experiment with methanol pulses of 0.5% and 1% (v/v). A, (continuous line), oxygen uptake rate OUR; (diamond) biomass dry cell weight concentration; B, (continuous line), carbon dioxide emission rate CER; (circle), calculated specific substrate uptake rate q_s_; (triangle up), calculated specific HRP productivity q_p_. Click here for file
