# Supplementary figures and images for: Recombinant protein expression in Pichia pastoris strains with an engineered methanol utilization pathway
Source: Microb Cell Fact. 2012 Feb 13;11:22. doi: 10.1186/1475-2859-11-22 (PMC3295664; doi:10.1186/1475-2859-11-22)

DAS1

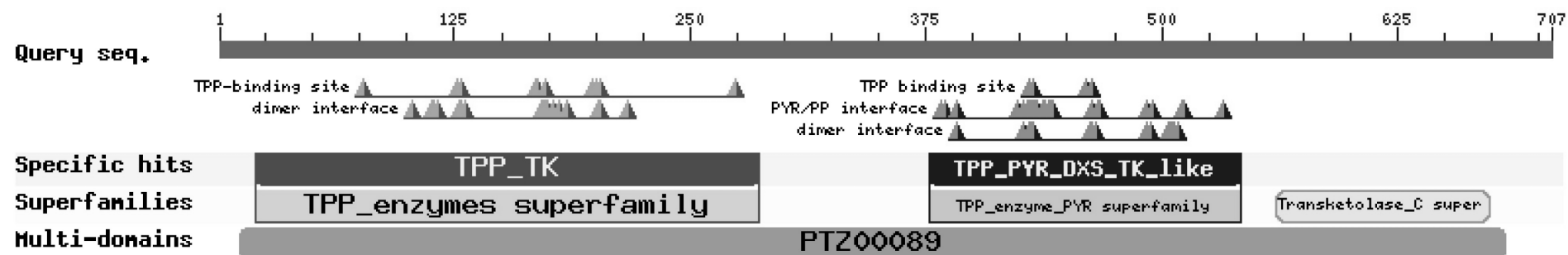

DAS2

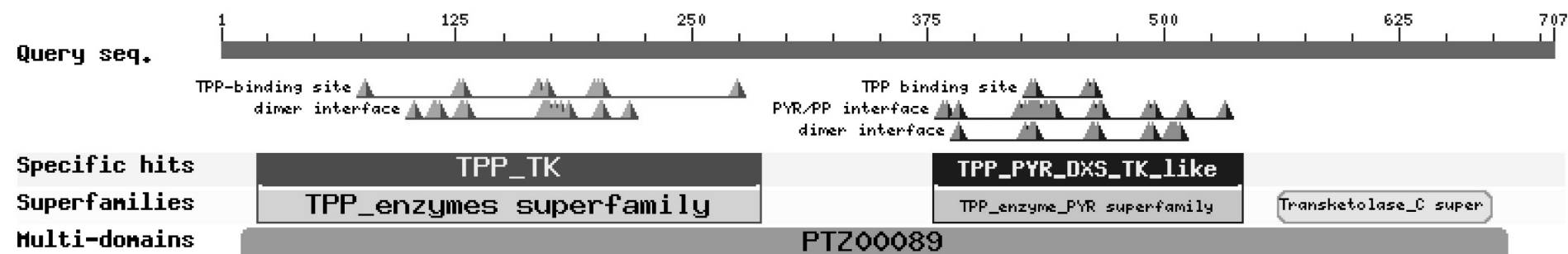

TKL1

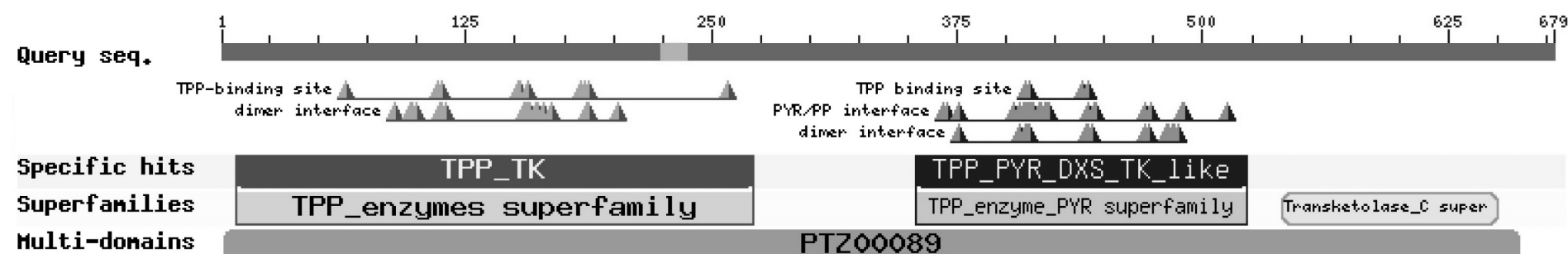

Supplement: Additional file 1 — Figure S1. Domain structure of DAS1, DAS2 and TKL1 (conserved domain prediction via CD-search tool [43-45]). All three enzymes belong to I, a thiamine pyrophosphate (TPP)-enzyme superfamily [NCBI CDD:cl01629]; II, a superfamily of TPP-depending enzymes containing a pyrimidine binding domain [NCBI CDD:cl11410]; and III, a superfamily of enzymes with a transketolase C-terminal domain [NCBI CDD:cl08363]. Amino acid sequences of P. pastoris strain CBS7435 from [16]. [file 1475-2859-11-22-S1.PDF]

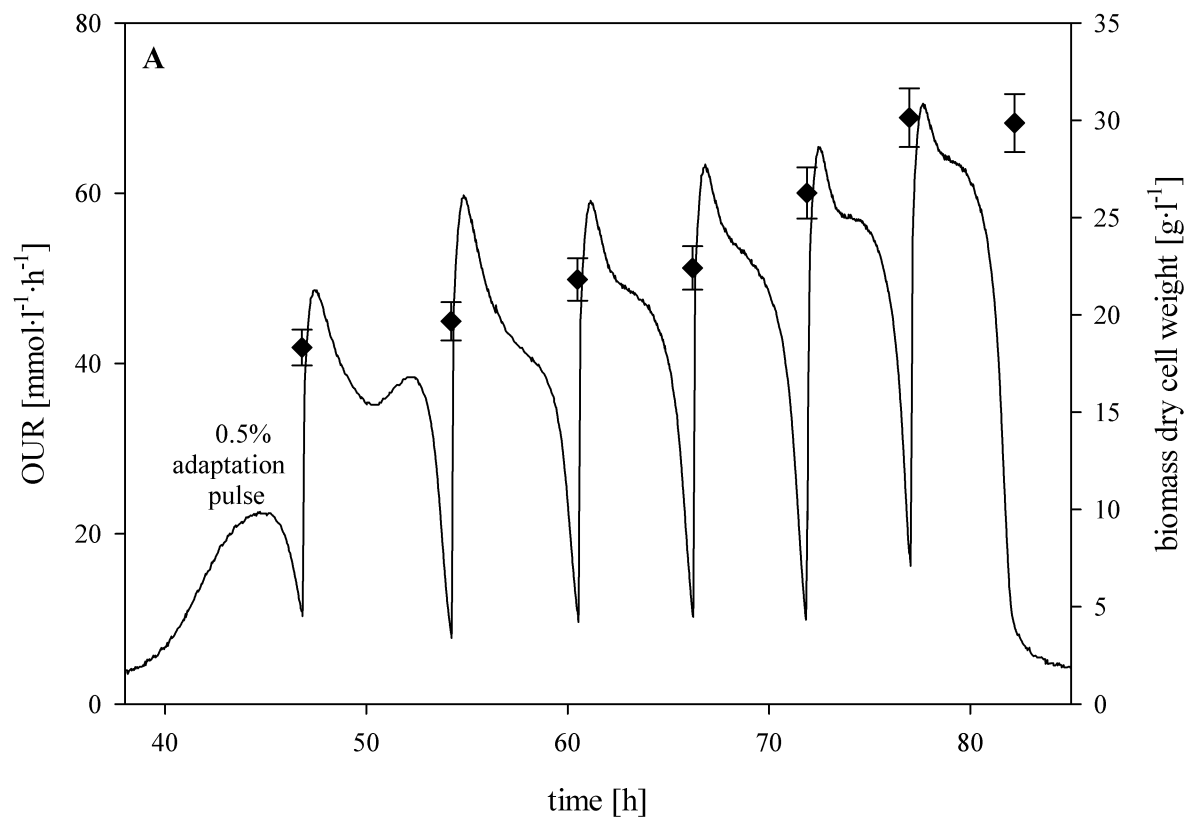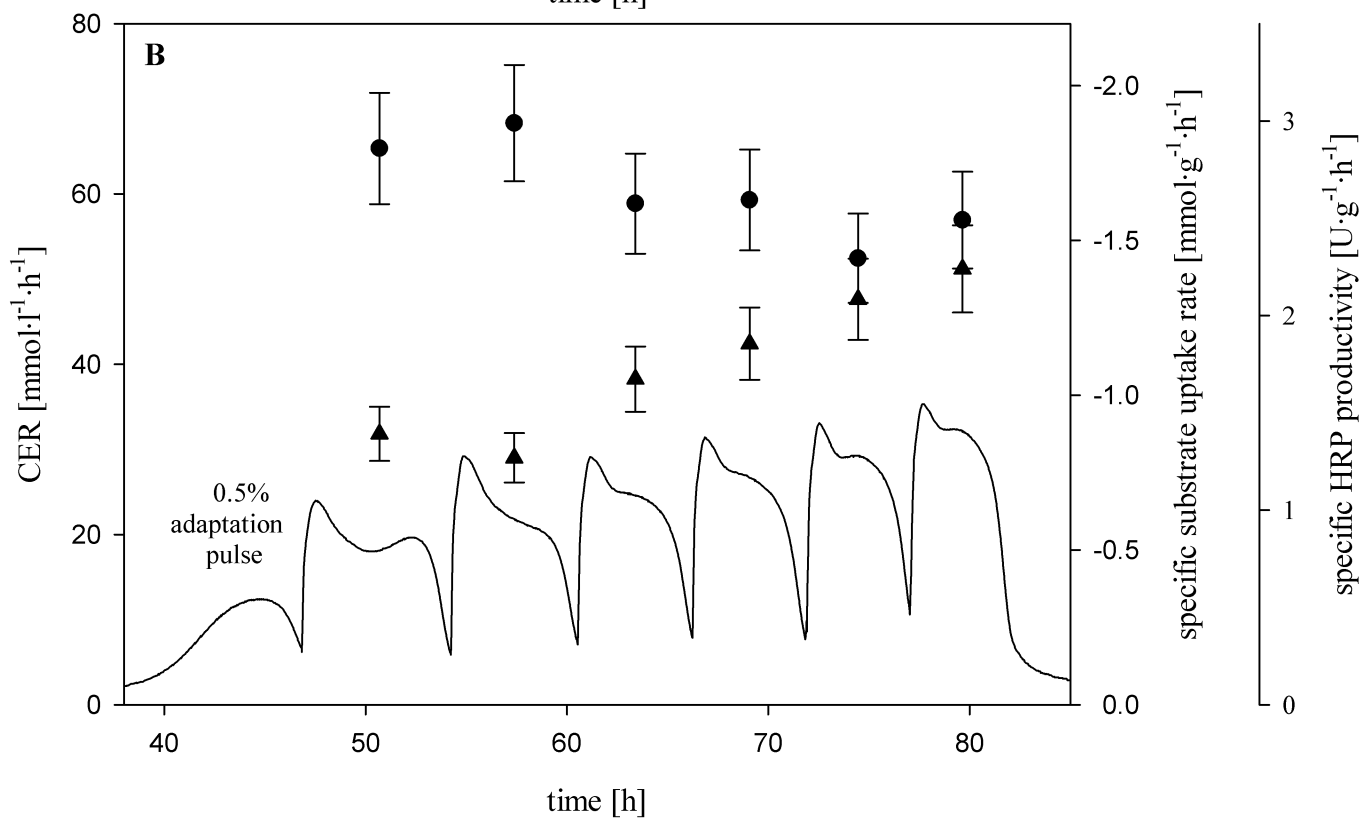

Supplement: Additional file 2 — Figure S2. Experimental strategy for the fast determination of strain specific parameters of the P. pastoris MutS HRP strain using a batch experiment with methanol pulses of 0.5% and 1% (v/v). A, (continuous line), oxygen uptake rate OUR; (diamond) biomass dry cell weight concentration; B, (continuous line), carbon dioxide emission rate CER; (circle), calculated specific substrate uptake rate qs; (triangle up), calculated specific HRP productivity qp. [file 1475-2859-11-22-S2.PDF]

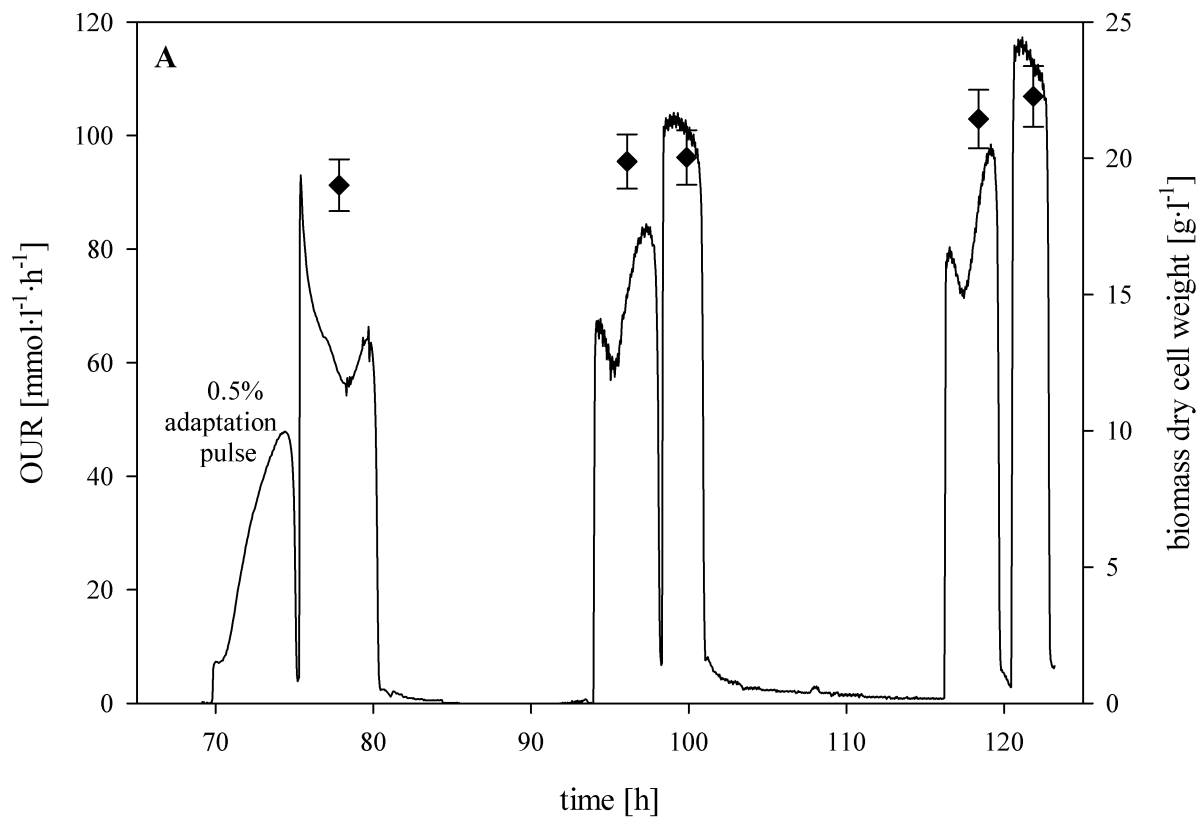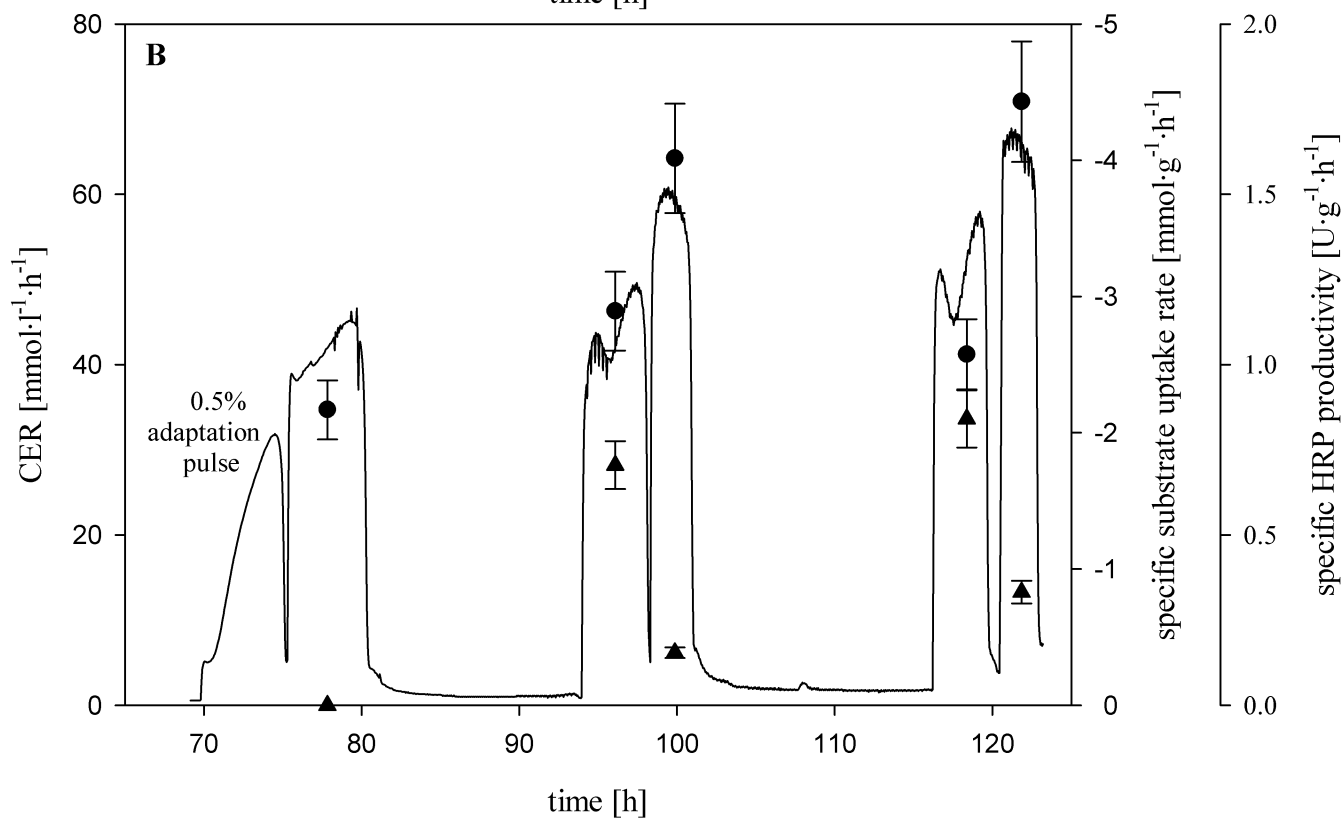

Supplement: Additional file 3 — Figure S3. Experimental strategy for the fast determination of strain specific parameters of the P. pastoris Mut+ HRP strain using a batch experiment with methanol pulses of 0.5% and 1% (v/v). A, (continuous line), oxygen uptake rate OUR; (diamond) biomass dry cell weight concentration; B, (continuous line), carbon dioxide emission rate CER; (circle), calculated specific substrate uptake rate qs; (triangle up), calculated specific HRP productivity qp. [file 1475-2859-11-22-S3.PDF]
